# Comparison of the association between different dietary pattern scoring indices and periodontitis and their population heterogeneity

**DOI:** 10.3389/fnut.2025.1590694

**Published:** 2025-06-19

**Authors:** Rui Zhang, Zushan Zhang, Shuming Ji, Wenjie Yang, Tengfei Long

**Affiliations:** ^1^Department of Stomatology, The Central Hospital of Wuhan, Tongji Medical College, Huazhong University of Science and Technology, Wuhan, Hubei, China; ^2^School & Hospital of Stomatology, Wuhan University, Wuhan, Hubei, China; ^3^Department of Clinical Research Management, West China Hospital, Sichuan University, Chengdu, China; ^4^Public Health Department, Wuhan Third Hospital, Tongren Hospital of Wuhan University, Wuhan, Hubei, China

**Keywords:** periodontitis, dietary pattern scoring index, restricted cubic splines, dietary intervention, population heterogeneity

## Abstract

**Objectives:**

Association between different dietary indices and periodontitis remained unclear. This study aims to compare the associations of four commonly dietary indices (including Healthy Eating Index-2020, HEI-2020; alternative Mediterranean Diet Score, aMED; Dietary Approaches to Stop Hypertension, DASH; Dietary Inflammatory Index, DII) with the risk of periodontitis.

**Methods:**

A cross-sectional study was designed using a publicly available data collected from the National Health and Nutrition Examination Survey (NHANES) between 2009 and 2014 (*N* = 8,571 adults over 30 years). After adjusting for confounders, dietary indices were included in logistic regression models by single, double and overall forms to explore the association with periodontitis. Odds ratios (ORs) for the dietary indices were adjusted by one-fourth of their scoring range to compare the effect sizes; and diminishing marginal receiver operating characteristic (ROC) curves analysis with univariate exclusion in the overall model was used to compare the contribution of the dietary indices to periodontitis. Restricted cubic splines (RCS) was used to explore the non-linear association in both the total population and various sub-populations.

**Results:**

Although all dietary indices exhibited a significant effect on periodontitis in single exposure model; only DASH and DII retained complete significance in the double exposure condition. In the overall model, aMED and DASH presented significantly positive associations, the corresponding OR were 1.147 (95%CI: 1.002–1.313) and 1.310 (95%CI: 1.139–1.507); but DII showed a negative association with OR of 0.675 (95%CI: 0.597–0.763). The ROC analyses showed that the contribution of dietary indices to periodontitis was second only to sex and ethnicity. The non-linear tests showed an approximately linear association for HEI-2020, aMED, and DASH, but a significant non-linear association for DII (*p* = 0.024). Subgroups of females, younger than 50 years old, non-Hispanic White, smokers, and the ratio of family income to poverty ≤ 2.4 were more consistent with the association found in the total population.

**Conclusion:**

A poor habit for DASH was robustly linked to the occurrence of periodontitis, while the other three dietary patterns were not. Our research suggests that including the DASH index in the evaluation of periodontitis risk and implementing targeted prevention strategies may be beneficial.

## Introduction

Periodontitis is a chronic inflammatory disease affecting the supporting structures of teeth (1). Severe periodontitis can lead to progressive destruction of the tooth supporting apparatus (such as gingiva, cementum, periodontal ligament, and alveolar bone) and eventual tooth loss, with profound impacts on masticatory function, aesthetics, and quality of life (2). According to the report of World Health Organization (WHO), as the sixth most prevalent disease globally, nearly 19% of the global adult population has severe periodontal disease, with more than 1 billion cases worldwide (3). In the United States and Europe, the combined direct (treatment costs) and indirect economic (due to loss in productivity) burdens of periodontal diseases are estimated at $154.06 billion and €158.64 billion, respectively (4).

Epidemiologically, periodontitis is intricately linked to systemic conditions, including cardiovascular disease, type 2 diabetes, obesity, rheumatoid arthritis, and Alzheimer’s disease, among others (5). These associations may arise from bidirectional mechanisms, that is the low-grade systemic inflammation driven by periodontal pathogens exacerbates metabolic dysregulation, while systemic diseases like diabetes amplify periodontal tissue destruction through heightened inflammatory burden and microbial dysbiosis (6, 7). Notably, smoking, poor oral hygiene, and socioeconomic disparities could further compound disease risk. This interaction between oral and systemic health underscores the urgency of identifying novel modifiable risk factors to disrupt the cycle of inflammation and disease progression, such as dietary patterns (8, 9).

Several pieces of evidence indicate the important role of dietary patterns in increasing the risk of chronic diseases through inflammatory regulation (10). Pro-inflammatory diets characterized by refined sugars and saturated fats demonstrate significant associations with heightened risks of type 2 diabetes mellitus, atherosclerosis, colorectal carcinoma, and periodontitis (11). Conversely, anti-inflammatory dietary components including vitamin C/E, *ω*-3 polyunsaturated fatty acids, and dietary fiber exhibit protective effects against Alzheimer’s disease progression, insulin resistance, and periodontal attachment loss (12–14). Notably, a dose–response relationship between dietary inflammatory index scores and periodontitis incidence has been validated through longitudinal cohort studies (15). Despite growing recognition of diet-periodontitis links, inconsistencies persist regarding which dietary components or composite indices most robustly predict disease risk, highlighting the need for standardized dietary assessment frameworks.

To address this gap, epidemiologic studies increasingly employ validated dietary pattern scoring systems. Among them, the Healthy Eating Index-2020 (HEI-2020) quantifies adherence to the Dietary Guidelines for Americans (DGA), 2020–2025, emphasizing vegetables, fruits, whole grains, dairy, protein foods (16). The alternative Mediterranean Diet Score (aMED) evaluates adherence to the Mediterranean diet, prioritizing plant-based foods, fish, and olive oil (17). The Dietary Approaches to Stop Hypertension (DASH) score, designed to reduce blood pressure, emphasizes low sodium, high potassium, and fiber intake (18). Lastly, the Dietary Inflammatory Index (DII) quantifies the inflammatory potential of diets based on pro-and anti-inflammatory nutrient profiles (19). While these indices are widely used in chronic disease research, their comparative utility in the risk of periodontitis remains underexplored.

This study aims to evaluate and compare the associations between four dietary pattern scoring indices (HEI-2020, aMED, DASH, and DII) and the risk of periodontitis. Additionally, our study also tries to compare the importance of dietary pattern scoring indices in influencing factors related to periodontitis, and evaluate the heterogeneity of the association in populations with different characteristics. Through this work, we seek to offer tailored dietary pattern recommendations for preventing of periodontitis.

## Materials and methods

### Study design and population

A cross-section study was designed based on the publicly available data from the National Health and Nutrition Examination Survey (NHANES) collected between 2009 and 2014, focusing on adults aged 30 years and older (20), as standardized periodontal examinations in NHANES are performed for this age group. Data collection occurred in two main phases. Initially, household interviews provided detailed demographic characteristics; including age, sex, ethnicity, educational degree, smoking status, and family income-to-poverty ratio (IPR) (21), and self-reported medical history, including hypertension, diabetes mellitus, chronic kidney diseases (CKD), cardiovascular diseases (CVD). Subsequently, participants underwent comprehensive clinical examinations at mobile centers included measurements of body mass index (BMI), blood pressure, fasting plasma glucose, and full-mouth periodontal assessments using clinical attachment loss (CAL) and probing depth (PD) at six sites per tooth.

Dietary intake was evaluated by using a two-step 24-h dietary recall procedure, with the first recall was performed at the time of examination, and the second was conducted via telephone 3–10 days later (22). These data were used to calculate four dietary indices, including HEI-2020, aMED, DASH, and DII, each reflecting distinct nutritional patterns linked to chronic disease risk.

**Figure 1:**
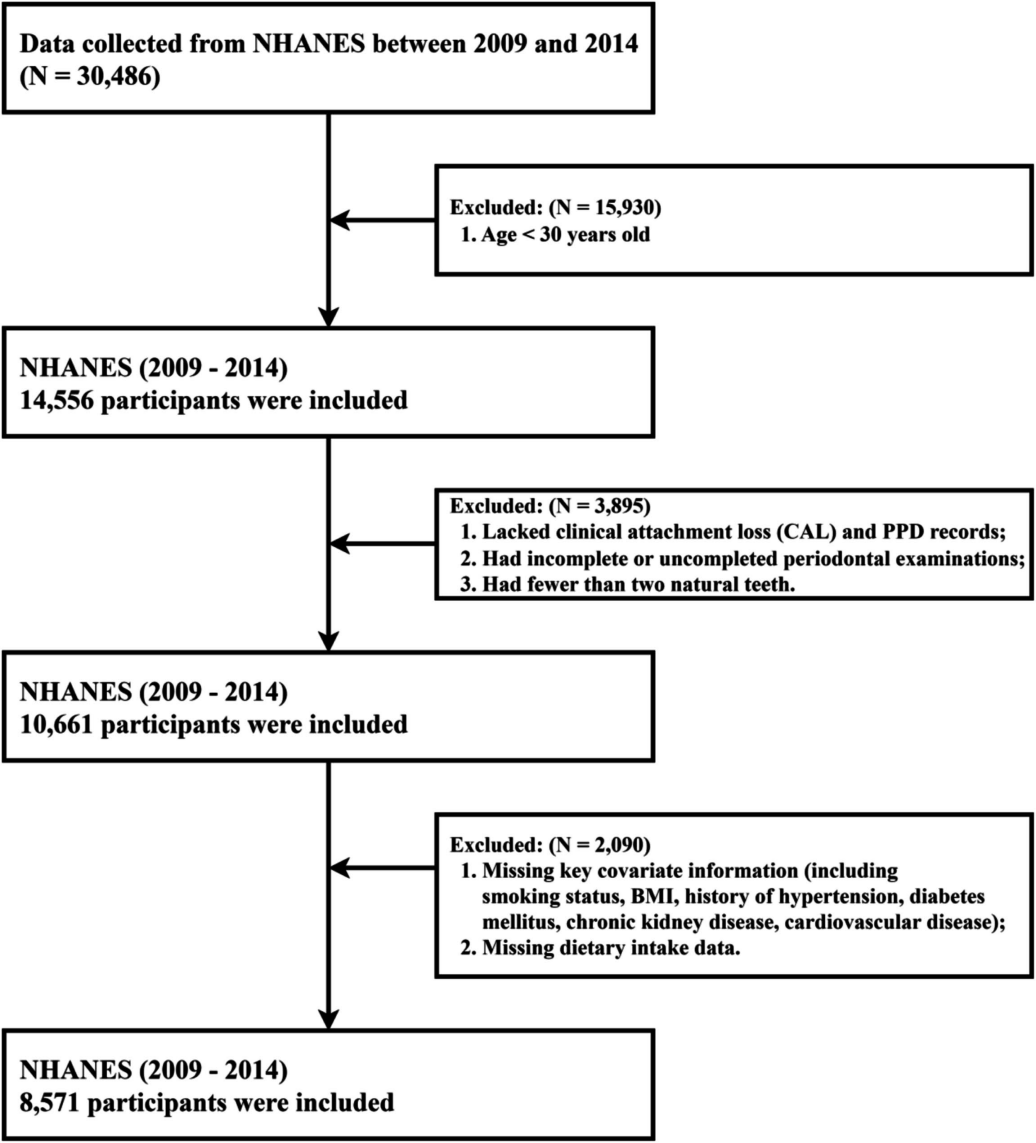
The study’s flow chart for the inclusion and exclusion of participants.

Of the 30,486 participants in the original survey, those aged ≥ 30 years old, had complete demographic data (including age, sex, ethnicity, educational degree, IPR), available information on key covariates (including smoking status, BMI, missing teeth count, and histories of hypertension, diabetes, CKD, or CVD), and valid dietary data from both recalls were enrolled in our study. Exclusion criteria included age < 30 years or incomplete demographic data (*n* = 15,930), incomplete or invalid periodontal assessments (including lacking records of CAL and PD, fewer than 2 natural teeth; *n* = 3,895), and missing critical covariate information or dietary recall data (*n* = 2,090). Eight thousand, five hundred seventy-one adults met all criteria and formed the final analytical sample, with the study’s flow chart shown in Figure 1. As NHANES data are de-identified and available through public repositories, ethical approval was not required beyond the program’s institutional review processes.

### Dietary pattern assessment

Our study employed four commonly utilized dietary pattern scoring indices, were the HEI-2020, aMED, DASH, and DII (23). Among these, HEI-2020 is the most recent version of the Healthy Eating Index (HEI), which was reviewed, updated, and developed based on the DGA (version of 2020–2025) (16). The HEI-2020 consists of 13 dietary components, covering aspects such as vegetables, fruits, whole grains, dairy, protein foods (e.g., meat, legumes), and fat intake (e.g., monounsaturated fats, saturated fats). Each dietary component is assigned a score ranging from 0 to 10 or 0 to 5 based on the difference between actual intake and recommended intake levels; and the total score of HEI-2020 sums to 100 points. HEI-2020 is an excellent tool for comprehensively assessing whether a diet aligns with health dietary recommendations, with higher scores indicating closer adherence to healthy eating standards. HEI-2020 has been widely applied in epidemiological research, particularly in evaluating dietary quality in relation to chronic diseases such as cardiovascular disease and diabetes.

The aMED is an alternative Mediterranean diet scoring index that simplifies and adjusts the core components of the Mediterranean diet (MED), such as vegetables (excluding potatoes and French fries), fruits, nuts, whole grains, legumes, fish, the ratio of monounsaturated to saturated fats, red and processed meats, and alcohol. The aMED is usually used to assess the degree of adherence to the Mediterranean diet in individual dietary patterns (17). Participants receive one point for each category if their intake exceeds the median, otherwise, they receive zero point. The scoring is reversed for red and processed meats as well as alcohol, where those below the median receive one point and those above receive zero point. The total score of aMED ranges from 0 to 9, with higher scores indicating closer alignment with the Mediterranean diet. Due to its simplified scoring method, aMED has been widely used in large-scale epidemiological studies. Currently, aMED is primarily employed to investigate the impact of Mediterranean dietary patterns on chronic diseases, particularly cardiovascular diseases.

The DASH index was designed to prevent and control hypertension. It focuses on eight key components: high intake of fruits, vegetables, nuts, legumes, low-fat dairy products, and whole grains, while promoting low intake of sodium, sweetened beverages, and red and processed meats (18). The DASH score is evaluated based on the quintiles of intake in the surveyed population, with each component assigned a score ranging from 1 to 5. Thus, the total DASH score ranges from 8 to 40. The score is particularly influenced by the intake of key minerals, such as sodium, potassium, calcium, and magnesium, meaning that diets with higher scores typically exhibit low sodium and high potassium, calcium, and magnesium levels. The DASH index is specifically designed to reduce the risk of hypertension, making it particularly useful for assessing dietary patterns related to blood pressure. It is widely applied in the prevention and treatment of hypertension and is also valuable for studies related to cardiovascular diseases.

The DII is a scoring tool designed to assess the potential of a diet to influence systemic inflammation (19). It evaluates the impact of various food components, including beneficial nutrients such as antioxidants, vitamins, and minerals, as well as pro-inflammatory factors such as sugars, saturated fats, and red meat. The calculation progress of the DII is relatively complex, which assigning scores based on the inflammatory potential of foods. DII incorporates 45 food components known to be associated with inflammation, and each food component’s intake is compared with the global average intake, and a score is assigned based on its global impact on inflammation. Generally, a positive score indicates pro-inflammatory foods (e.g., red meat, high sugar, and high-fat foods), while anti-inflammatory foods (e.g., fruits, vegetables, and whole grains) receives a negative score. Based on global DII simulations, the score range typically spans from −9 (strongly anti-inflammatory) to +8 (strongly pro-inflammatory). DII is primarily used to assess the impact of diet on chronic inflammation, which is closely linked to several chronic diseases, including cardiovascular diseases, diabetes, and cancer.

### Outcome definitions

Among all the NHANES participants, adults aged 30 years and older, who had at least one natural tooth, were eligible for a full-mouth periodontal examination (FMPE), which included the assessment of gingival recession and probing pocket depth (PPD) measures (14,556 individuals). According to the standards provided by the Centers for Disease Control and Prevention (CDC) and the American Academy of Periodontology (AAP), participants were excluded in order to ensure a complete diagnosis of periodontitis if they lacked clinical attachment loss (CAL) and PPD records, had incomplete or uncompleted periodontal examinations, or had fewer than two natural teeth (10,661 individuals) (24). All dental examiners were trained and calibrated by the reference examiners of the survey.

Severe periodontitis is defined as CAL of at least 5 mm in the interproximal regions of at least two non-adjacent teeth, with PPD of at least 6 mm in the interproximal region of at least one tooth. Moderate periodontitis is defined as the presence of PPD greater than or equal to 5 mm in two or more non-adjacent teeth’ interproximal regions, or CAL greater than or equal to 3 mm in the interproximal regions of two or more non-adjacent teeth. Participants diagnosed with mild periodontitis must exhibit a CAL of at least 1 mm in the interproximal regions of at least two non-adjacent teeth, or a PPD greater than or equal to 4 mm in the interproximal regions of at least two or more non-adjacent teeth (25, 26). All cases of periodontitis diagnosed as mild, moderate, or severe based on CAL in the interproximal regions and PPD had to meet the criteria of at least two non-adjacent teeth with a CAL larger than 3 mm. In this study, we categorized mild, moderate, and severe periodontitis into one group (having periodontitis) and the other group as having no periodontitis.

### Covariates assessment

To enhance the precision of exploring associations between dietary pattern scoring indices and periodontitis, a comprehensive set of covariates was selected for model adjustment, including demographic characteristics, lifestyle factors (smoking history), and health status (hypertension and diabetes). Demographic variables, derived from household questionnaires, included age, sex, ethnicity, educational degree, and the family IPR. BMI was calculated from measured height and weight during physical examinations and categorized into four groups per international guidelines: underweight (<18.5 kg/m^2^), normal weight (18.5–25 kg/m^2^), overweight (25–30 kg/m^2^), and obesity (>30 kg/m^2^) (27). Smoking history was dichotomized into individuals with a history of smoking (current or former smokers) and never-smokers.

Hypertension was defined as either self-reported diagnosis during household interviews or laboratory-based criteria (systolic blood pressure ≥130 mmHg or diastolic blood pressure ≥80 mmHg) (28). Diabetes mellitus was identified through self-reported diagnosis or laboratory-confirmed thresholds [fasting plasma glucose ≥7.0 mmol/L or glycated hemoglobin (HbA1c) ≥ 6.5%] (29). The CKD status was diagnosed through standardized biochemical and urinary examinations. eGFR was calculated according to the CKD Epidemiology Collaboration (CKD-EPI) equation, with CKD diagnosis defined as either (a) eGFR <60 mL/min/1.73 m^2^ or (b) ACR > 30 mg/g. CVD was obtained according to the reported or self-admitted physician diagnoses. Self-admitted CVD was assessed by asking the following questions: “Has a doctor or other health professional ever told you that you have a heart attack/coronary heart disease/angina/congestive heart failure/stroke?.” The answer could be “Yes/No/did not know,” and “Yes” was considered as a CVD patient; the “did not know” participants would be excluded. These covariates were rigorously operationalized to account for potential confounding effects and align with standardized diagnostic criteria, ensuring robustness in evaluating the independent relationship between dietary patterns and periodontitis.

### Statistical analysis

Participants were classified into periodontitis and non-periodontitis groups based on the results of clinical periodontal examinations. Descriptive analysis was employed to summarize the baseline characteristics of the study population, which included age, sex, ethnicity, educational degree, family IPR, BMI, smoking history, hypertension diabetes, and CVD, as well as dietary pattern scoring indices. Continuous variables were expressed as means ± standard deviations, whereas categorical variables were presented as frequencies and percentages. In the comparison between groups, independent t-tests or Mann–Whitney *U* tests were used for continuous variables, and chi-square tests were used for categorical variables.

For the purpose of clarity, we performed reverse calculations for HEI-2020, DASH, and aMED (that is, subtracting the actual score from the total score of each index) to align with the concept that a higher score indicates a less healthy diet; which does not apply to the nonlinear trend analysis section. After controlling for demographic characteristics and comorbidities, we utilized three types of multiple logistic regression models to examine the relationships between four dietary pattern scoring indices (HEI-2020, aMED, DASH, DII) and periodontitis. In details, (1) four single-diet models, each including only one dietary pattern index, were independently used to evaluate the association between each dietary pattern and periodontitis; (2) for each dietary pattern index, three double-diet models were constructed by adjusting for the other dietary patterns one by one; (3) an overall model including all four dietary patterns were established to assess the associations when controlling all the other three patterns. Association estimates were standardized based on one-fourth of each index’s scoring range to ensure comparability. Model coefficients and odds ratios (ORs) with 95% confidence intervals (CIs) were visualized through forest plots, including an overall composite model visualization. Risk assessment was quantified via receiver operating characteristic (ROC) curve analysis, calculating marginal area under the curve (AUC) differences between the full model and reduced models that systematically excluded individual covariates or the dietary indices collectively, enabling comparative assessment of the effect strength among different variables (30).

The restricted cubic splines (RCS), with the Akaike information criterion (AIC) optimization program used to select the most suitable knots; were applied to explore any potential non-linear dose–response relationships between different dietary indices and periodontitis in the total population and various sub-populations, thereby facilitating the visualization and testing of dose–response trends between dietary pattern scoring index and the risk of periodontitis (31). Results are presented as ORs with 95% CIs to quantify the strength and direction of the associations. All statistical analyses were performed using R version 4.4.1, with a two-tailed *p-*value ≤ 0.05 considered statistically significant.

## Results

This study included 8,571 individuals with an average age of 52.131 ± 14.122 years. Among them, 5,439 individuals (63.46%) were diagnosed with periodontitis, and 3,132 individuals (36.54%) were without periodontitis. Table 1 displays the baseline characteristics of the study population categorized by periodontitis status. In particular, individuals who are male, older, Mexican American, have lower education levels, obesity, smoking habits, lower family IPR, hypertension, diabetes, CKD and CVD are more likely to suffer from periodontitis (*p* < 0.001). For dietary patterns, individuals with periodontitis have lower scores for HEI-2020, and aMED, DASH than those without periodontitis. No statistically significant difference was observed for DII (*p* = 0.502).

**Table 1:** Description of demographic characteristics and associated factors for all participants divided by whether confirmed as periodontitis.

Variables	Overall (*N* = 8,571, 100%)	Without periodontitis (*N* = 3,132, 36.542%)	With periodontitis (*N* = 5,439, 63.458%)	*P*-value
Sex				<0.001
Male	4,166 (48.606)	1,179 (37.644)	2,987 (54.918)	
Female	4,405 (51.394)	1953 (62.356)	2,452 (45.082)	
Age	52.131 ± 14.122	50.711 ± 14.383	52.949 ± 13.905	<0.001
Ethnicity				<0.001
Mexican American	1,220 (14.234)	283 (9.036)	937 (17.227)	
Other Hispanic	849 (9.905)	322 (10.281)	527 (9.689)	
Non-Hispanic White	3,901 (45.514)	1720 (54.917)	2,181 (40.099)	
Non-Hispanic Black	1,690 (19.718)	472 (15.070)	1,218 (22.394)	
Other Race	911 (10.629)	335 (10.696)	576 (10.590)	
Educational degree				<0.001
Less than 9th grade	753 (8.785)	178 (5.683)	575 (10.572)	
9–11th grade	1,088 (12.694)	294 (9.387)	794 (14.598)	
High school graduate	1851 (21.596)	609 (19.444)	1,242 (22.835)	
Some college or AA	2,467 (28.783)	914 (29.183)	1,553 (28.553)	
College graduate	2,412 (28.141)	1,137 (36.303)	1,275 (23.442)	
BMI Level				<0.001
Underweight or Normal	2,216 (25.855)	913 (29.151)	1,303 (23.957)	
Overweight	2,976 (34.722)	1,068 (34.100)	1908 (35.080)	
Obesity	3,379 (39.424)	1,151 (36.750)	2,228 (40.963)	
Smoking History	3,712 (43.309)	1,147 (36.622)	2,565 (47.159)	<0.001
Family IPR	2.701 ± 1.658	3.018 ± 1.657	2.518 ± 1.630	<0.001
Hypertension	4,452 (51.943)	1,459 (46.584)	2,993 (55.028)	<0.001
Diabetes Mellitus	1,447 (16.883)	431 (13.761)	1,016 (18.680)	<0.001
Chronic kidney disease	1,318 (15.377)	413 (13.186)	905 (16.639)	<0.001
Cardiovascular disease	703 (8.202)	206 (6.577)	497 (9.138)	<0.001
HEI-2020 index	53.094 ± 12.032	54.375 ± 12.182	52.356 ± 11.883	<0.001
aMED index	3.612 ± 1.397	3.785 ± 1.438	3.512 ± 1.362	<0.001
DASH index	22.925 ± 5.086	23.785 ± 5.148	22.429 ± 4.983	<0.001
DII index	2.316 ± 2.082	2.336 ± 2.131	2.305 ± 2.054	0.502

Independent Student-*t* test was used to test the difference between periodontitis and non-periodontitis groups for continuous variables. Chi-square test was used to test the difference for categorical variables. Some college or AA, some college or associates (AA) degree; BMI, Body Mass Index; Family IPR, the ratio of family income to poverty. The ranges of BMI for underweight, normal, overweight and obesity were <18.5, 18.5–24.9, 25.0–29.9, and ≥30 kg/m^2^, respectively.

**Figure 2:**
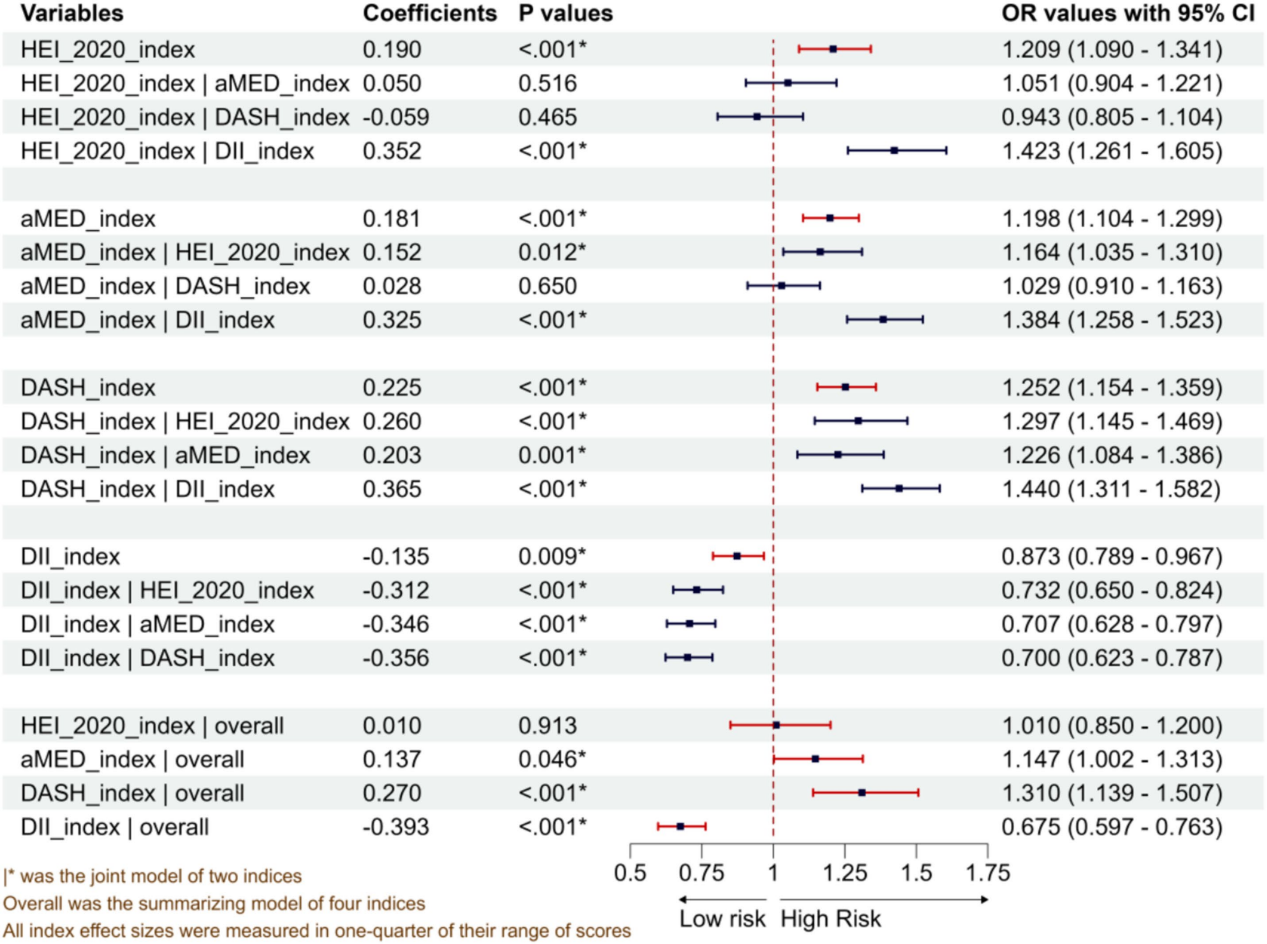
Associations between four dietary pattern scoring indices and periodontitis from single-diet, double-diet, and four-diet models.

As shown in Figure 2, the multiple logistic regressions with a single dietary pattern, after adjusting for potential confounding factors, revealed that HEI-2020, aMED, and DASH were positively associated with periodontitis. The ORs for a one-quarter increase in the maximum score range were 1.209 (95% CI: 1.090–1.341), 1.198 (95% CI: 1.104–1.221), and 1.252 (95% CI: 1.154–1.359) for the three patterns, respectively. However, DII presented a negative association with OR of 0.873 (95% CI: 0.789–0.967). The double-dietary-patterns model showed that DASH and DII still exhibited statistically significantly positive and negative associations, respectively, when adjusting for other dietary patterns. However, HEI-2020 exhibited insignificant associations when adjusting for either aMED or DASH; aMED exhibited insignificant associations when adjusting for DASH. The full logistic regression with four dietary patterns supported the results, i.e., statistically significantly positive association was found for aMED and DASH and negative association for DII, as well as insignificant associations for HEI-2020.

**Figure 3:**
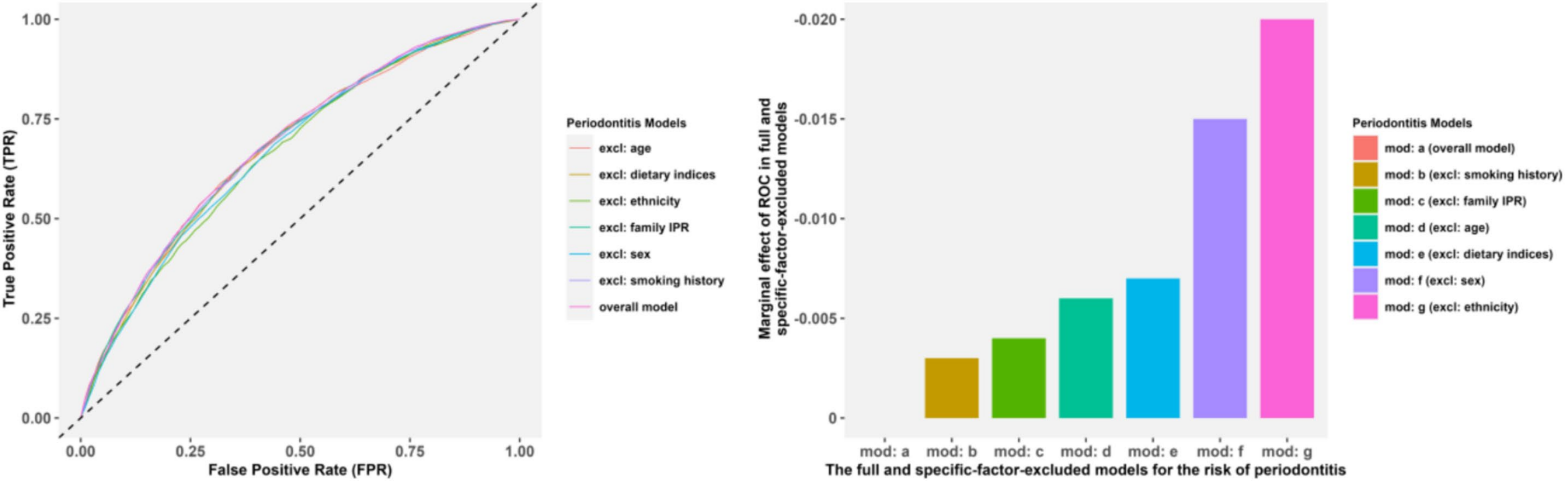
The ROC and AUC comparisons in the full model and the specific-factor-excluded models. Panels **(A,B)** presented the ROC and AUC comparisons, respectively.

The variance inflation factors in the overall model for the four dietary pattern scoring indices were 3.234, 3.297, 3.703 and 1.703, respectively, indicating low multiple collinearities. As shown in Table 2 and Supplementary Figure 1, after these covariates were adjusted for each other, the full model indicated that sex (OR = 0.540), ethnicity, educational degree (the OR of college education = 0.689), smoking history (OR = 1.290), increasing age (OR = 1.017/year), and higher family IPR (OR = 0.894) were still significant predictors for periodontitis (*p* < 0.05). However, the statistical significance for BMI, hypertension, diabetes mellitus, CKD and CVD was not maintained. The results of ROC curves revealed a stable ROC performance across all the specific-factor-excluded model, with the most downward for ethnicity, sex and dietary patterns removed models, aligning with their substantial changes in AUC. Overall, ethnicity, sex and dietary patterns contributed the most to whether an individual has periodontitis, followed by age, family IPR and smoking history, with details shown in Figure 3.

**Table 2:** Multivariate logistic regression results of the effects of all dietary patterns scoring indices on periodontitis.

Variables	Coefficients	Std. error	*Z*-value	OR	95%CI of OR		*P*-value
					Lower	Upper	
Sex (Ref = male)	−0.617	0.052	−11.952	0.540	0.488	0.597	<0.001*
Age	0.017	0.002	8.382	1.017	1.013	1.021	<0.001*
Ethnicity (Ref = Mexican American)							
Other Hispanic	−0.607	0.103	−5.871	0.545	0.445	0.667	<0.001*
Non-Hispanic White	−0.857	0.087	−9.893	0.424	0.358	0.502	<0.001*
Non-Hispanic Black	−0.192	0.096	−1.997	0.825	0.683	0.996	0.046*
Other Race	−0.253	0.109	−2.319	0.777	0.627	0.961	0.020*
Educational degree (Ref = Less than 9th grade)							
9–11th grade	−0.015	0.118	−0.124	0.986	0.782	1.240	0.901
High school graduate	−0.157	0.111	−1.417	0.855	0.688	1.061	0.157
Some college or AA	−0.196	0.109	−1.795	0.822	0.663	1.017	0.073
College graduate	−0.372	0.116	−3.202	0.689	0.548	0.865	0.001*
BMI level (Ref = underweight or normal)							
Overweight	0.023	0.063	0.362	1.023	0.905	1.157	0.717
Obesity	0.108	0.064	1.670	1.114	0.981	1.264	0.095
Smoking history	0.255	0.050	5.053	1.290	1.169	1.425	<0.001*
Family IPR	−0.112	0.017	−6.693	0.894	0.865	0.924	<0.001*
Hypertension	0.055	0.054	1.025	1.056	0.951	1.173	0.306
Diabetes mellitus	−0.013	0.070	−0.188	0.987	0.860	1.133	0.851
Chronic kidney disease	0.057	0.072	0.793	1.059	0.919	1.221	0.428
Cardiovascular disease	−0.026	0.095	−0.279	0.974	0.810	1.175	0.780
HEI-2020 index	0.010	0.088	0.110	1.010	0.850	1.200	0.913
aMED index	0.137	0.069	1.996	1.147	1.002	1.313	0.046*
DASH index	0.270	0.071	3.783	1.310	1.139	1.507	<0.001*
DII index	−0.393	0.063	−6.262	0.675	0.597	0.763	<0.001*

*Presented as statistical significant; OR was the odds ratios in full model; for each dietary pattern scoring index, the coefficients, std. error, OR and 95%CI of OR were all estimated with the standardized per one-fourth of each index’s scoring range. Some college or AA, some college or associates (AA) degree; BMI, Body Mass Index; Family IPR, the ratio of family income to poverty.

**Figure 4:**
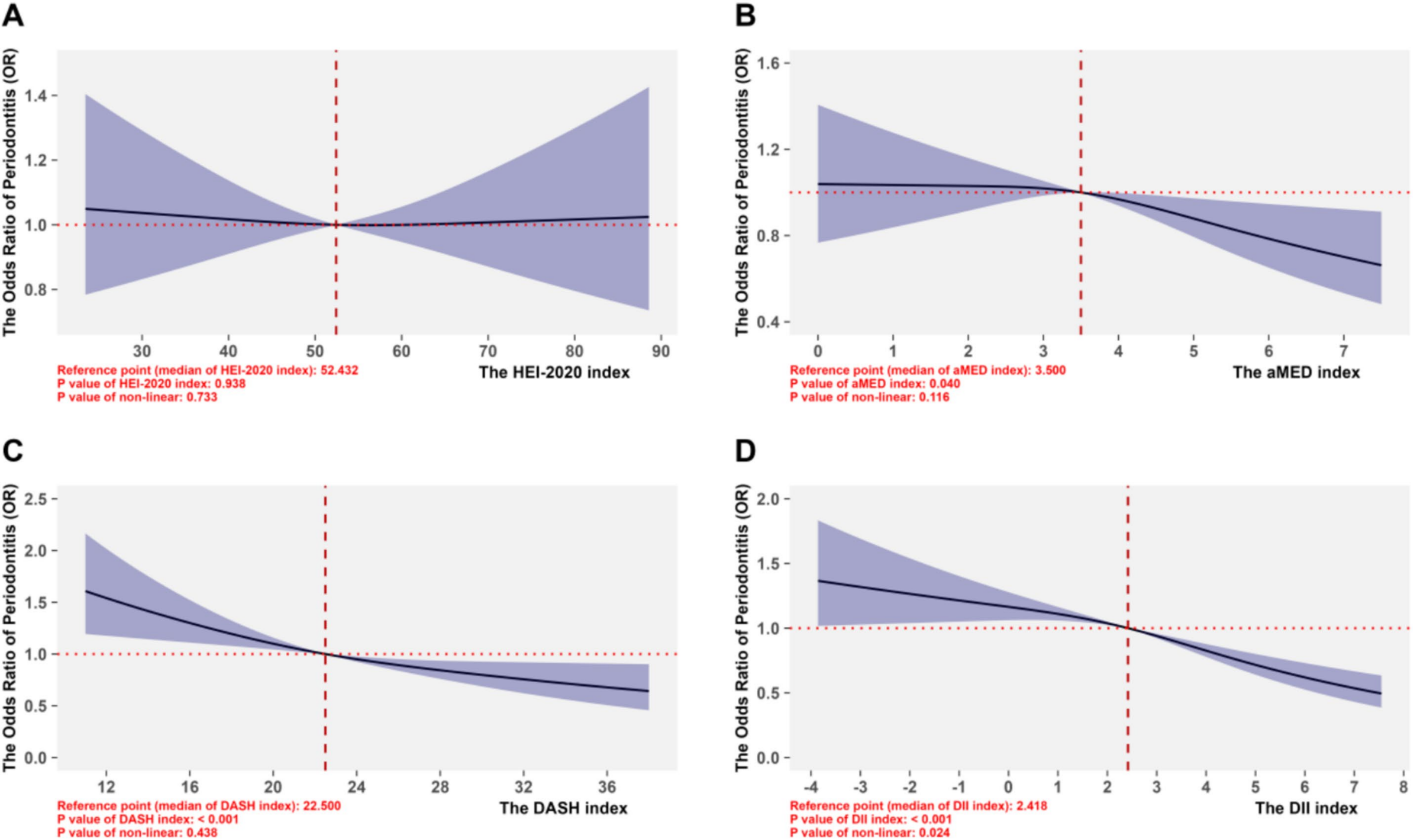
Non-linear associations between four dietary pattern scoring indices and periodontitis. **(A-D)** Corresponded to the RCS performances of the HEI-2020 index, aMED index, DASH index, and DII index, respectively, for the risk of periodontitis.

In the section of non-linear analyses, we replaced the dietary scores of HEI-2020, aMED, and DASH with their original true measurement scores; and evaluated the non-linear dose–response associations between dietary indices and the risk of periodontitis using RCS with knots optimized via AIC value, shown in Supplementary Figure 2. For all indices, the AIC were minimized in the model with 3 knots, and this configuration was retained for subsequent analyses. As shown in Figure 4, HEI-2020 showed little association with the risk of periodontitis; for the aMED index, using the median value (3.500) as the reference point, the negative dose–response association on periodontitis only significant when the scores exceeded the reference point; and for DASH with the reference point of 22.5, the ORs exceeded 1.0 at lower scores (8–22.5) but declined progressively, reaching values below 1.0 beyond 22.5 (up to 40), indicating reduced periodontitis risk with higher adherence. Although the non-linear trend test was statistically insignificant (*p* > 0.05), the overall inverse linear association of DASH remained robust. In contrast, the DII also exhibited a significantly negative association with the risk of periodontitis (*p* < 0.05), which higher scores indicates a more pro-inflammatory dietary pattern.

Furthermore, RCS analyses had also been used in subgroups to explore the population heterogeneity of the association between different dietary indices and periodontitis, with the results shown in Supplementary Figures 3–15. The results of subgroup analyses showed that the subgroups of females, younger than 50 years old, non-Hispanic White, smokers, and the ratio of family income to poverty ≤ 2.4 were more consistent with the association found in the total population (all subgroups were classified according to the categories of the categorical data or the median of the continuous data).

## Discussion

Our study was the first time to compare the association’s intensity of four commonly dietary pattern scoring indices with periodontitis, evaluate the importance of dietary patterns in factors related to periodontitis and explore its population heterogeneity. The single-diet models showed statistically significant positive associations for HEI-2020, aMED, and DASH, but a negative association for DII. After adjusting for other diet patterns, both double-diet and overall models revealed robust significant associations for DASH and DII, but not for aMED and HEI-2020. In the subgroups of females, younger than 50 years old, non-Hispanic White, smokers, and the ratio of family income to poverty ≤ 2.4, the performance of the associations for those dietary indices were more consistent with the associations found in the overall model. Given that a lower score for the HEI-2020, aMED and DASH, and a higher score for the DII indicates a poorer dietary habit, these results suggested that a poor habit related to DASH may be robust linked to the occurrence of periodontitis, whereas the other three dietary patterns were not. Our findings suggest that incorporating DASH into the risk assessment of periodontitis and optimizing relevant prevention strategies would be beneficial.

Our study revealed significant heterogeneity in the associated utility of dietary pattern scoring indices for periodontitis, with DASH and DII exhibiting robust associations. Among these dietary indices, the DASH diet, which selected as an example of healthy plant-based diets recommended by international (American Heart Association, AHA) and European guidelines (European Atherosclerosis Society, EAS), exhibited a protective dose–response association with the risk of periodontitis (32). This pattern characterized by sodium restriction, potassium optimization, and reduced saturated fat and intake, probably could mitigates endothelial dysfunction and enhances systemic antioxidant capacity, thereby attenuating periodontal oxidative stress (33). In contrast, the HEI-2020 showed limited utility, possibly due to its broad “adequacy-moderation” framework diluting these specific mechanisms (34). Similarly, the aMED also displayed limited significant association with periodontitis, especially in the lower score range; which was consistent with the findings of a Moroccan study (35); and in a cross-sectional survey study of 6,209 participants conducted by Altun et al. (32), DASH was also found to have a stronger association with periodontitis than aMED. Cultural and behavioral differences were the possible reasons for this result, especially the Mediterranean-centric components in MED index (e.g., olive oil, moderate wine) may lack specificity in modulating periodontal microbiota among non-Mediterranean populations (36).

The paradoxical inverse association between DII score and the risk of periodontitis (e.g., lower risk of periodontitis with higher pro-inflammatory scores) from our cross-sectional findings deserves scrutiny (37, 38). A Korean study based on the Korean Genome and Epidemiology Study Health Examinee cohort (KoGES_HEXA) explored the relationship between DII scores and periodontitis from both cross-sectional and longitudinal follow-up perspectives (39). This study found that the association between DII scores and periodontitis was not entirely significant from the perspective of cross-sectional survey (the risk of periodontitis between male participants with the DII score in Quartile 2 and Quartile 1: OR = 1.26, 95%CI: 0.99–1.62; the risk of periodontitis between female participants with the DII score in Quartile 4 and Quartile 1: OR = 1.13, 95%CI: 0.96–1.33), but were both significant in long-term follow-up, which was stronger than the cross-sectional evidence. In addition, a cross-sectional survey conducted by Syrjäläinen et al. (40), found that there was no difference in DII scores between participants with and without periodontitis, which was consistent with the findings of our study. Furthermore, we compared our results of the association between DII score and periodontitis with two studies which also based on NHANES (An Li’s study focused on patients with moderate to severe periodontitis, with mild periodontitis and non-periodontitis considered as one group) (41, 42). These studies both grouped the DII scores by tertiles (divided as low, medium and high group), and when compared to tertile 1, the significant increasing risk of periodontitis only was found in the group of tertile 3 in An Li’s study (adjusted OR in tertile 3 = 1.53, 95%CI: 1.33–1.77; adjusted OR in tertile 2 = 0.92, 95%CI: 0.80–1.06) (42); and found no significant association in Jie Feng’s study (adjusted OR in tertile 3 = 1.11, 95%CI: 0.98–1.25; adjusted OR in tertile 2 = 1.08, 95%CI: 0.96–1.21) (41), respectively.

According to the comparison with other studies, there are three hypotheses that may be able to explain the paradoxical association between DII and periodontitis. First, the recent inflammatory dietary patterns captured by the cross-sectional survey were not sufficient to directly induce clinical periodontitis. Second, existing periodontal inflammation in participants may prompt their dietary modifications (e.g., reduced red meat consumption due to chewing discomfort), creating reverse causation bias. Besides, systemic inflammatory biomarkers such as C-reactive protein (CRP) and IL-6 may not fully reflected the local periodontal inflammation, resulting in the inability of DII to sensitive feedback the risk of periodontitis.

Additionally, the differential associations observed among dietary indices may be partially explained by their distinct macronutrient profiles and their roles in periodontal pathogenesis. The DASH diet, rich in complex carbohydrates (whole grains, legumes), plant-based proteins (nuts, seeds, legumes), and unsaturated fats (low saturated fat, high nuts/seeds), likely exerts protective effects through anti-inflammatory and antioxidant pathways (32). For instance, its emphasis on potassium and magnesium supports endothelial function and reduces oxidative stress, which are critical in attenuating periodontal tissue destruction. In contrast, diets with lower DASH adherence may be higher in refined carbohydrates and sodium, promoting pathogenic bacterial proliferation (43). The aMED, while rich in monounsaturated fats (olive oil), may show limited relevance in non-mediterranean populations due to variations in lipid sources and cultural dietary practices. The DII, which correlates with pro-inflammatory components like saturated fats, *trans* fats, and sugar, showed a paradoxical inverse association in our cross-sectional analysis, possibly influenced by reverse causation (e.g., periodontal inflammation altering dietary choices) or the nuanced interaction between systemic macronutrient intake and local oral microbiota. Understanding these macronutrient-driven mechanisms allows for more precise dietary recommendations, such as reducing refined carbohydrates to limit bacterial glycolysis, increasing anti-inflammatory proteins (e.g., fish, legumes), and prioritizing unsaturated fats over saturated fats may collectively modulate host–microbe interactions and slow periodontal tissue degradation (15, 44).

On the other hand, the comparable importance of dietary indices and traditional risk factors (e.g., sex, age, ethnicity, smoking and family IPR) in influencing periodontitis underscores the underappreciated role of diet in periodontitis prevention. While traditional risk factors are either non-modifiable or inertia factors, dietary patterns offer actionable intervention targets. For instance, a quartile improvement in DASH adherence could theoretically offset 15 years of aging-related risk accumulation, which is a hypothesis requiring longitudinal validation. In general, the superior consistency of DASH in evaluating the risk of periodontitis within U. S. populations possibly stems from its alignment with hypertension prevention guidelines, which might overlap or cover part of the pathological mechanism of periodontitis.

The findings of this study hold substantial clinical relevance by identifying the DASH index as a robust predictor of periodontitis risk, particularly in subgroups such as females, younger adults, non-Hispanic White, smokers, and individuals with lower socioeconomic status (IPR ≤ 2.4). Clinically, integrating the DASH score into periodontal risk assessments could enable targeted interventions to improve dietary habits, especially given its alignment with established guidelines for hypertension and cardiovascular health. For example, patients with poor DASH adherence (characterized by high sodium intake, low fruit or vegetable consumption, and inadequate whole grains) may benefit from personalized nutrition counseling to reduce sodium, increase potassium-rich foods, and prioritize plant-based proteins, modifications that not only prevent periodontal inflammation but also mitigate comorbidities like hypertension. Public health initiatives could leverage these insights to design population-specific education programs, particularly in underserved groups where dietary risks for periodontitis are most pronounced. By emphasizing the actionable nature of dietary patterns, clinicians can empower patients to adopt preventive strategies that complement traditional treatments, potentially slowing disease progression and improving long-term oral health outcomes.

Despite its strengths, our study has several limitations inherent to its design and data source. First, as a cross-sectional analysis of NHANES data, causal inferences between dietary patterns and periodontitis risk cannot be established. Temporal relationships may be obscured by reverse causality, particularly for dietary index like the DII, where dietary modifications following periodontal symptoms could bias associations. Second, dietary intake was assessed using 24-h recalls, which are susceptible to recall bias and may not fully capture long-term dietary habits. Although NHANES employs rigorous protocols to enhance data accuracy, misclassification of dietary exposures remains a possibility. Third, we adjusted for a comprehensive set of confounders, but residual confounding from unmeasured factors (e.g., genetic predisposition, oral hygiene practices, or access to dental care) cannot be ruled out. Specifically, while dental floss and mouthwash use were partially recorded, their causal impact on periodontitis remains unclear, with low-quality evidence from existing reviews (45). Consequently, this study does not account for potential confounding by these behaviors. Future longitudinal research with consistent, detailed oral hygiene data is needed to validate how dietary patterns interact with oral health practices in influencing periodontitis risk. Finally, the generalizability of findings may be limited to the U. S. population, as NHANES sampling frames are nationally representative but may not reflect dietary patterns or periodontal disease profiles in other regions. Future longitudinal studies with repeated dietary assessments and standardized periodontal examinations are needed to validate these findings and elucidate causal pathways.

## Conclusion

A poor habit for DASH may be robust associated with the occurrence of periodontitis, while the other three dietary patterns were not. DASH is the most related dietary pattern index for evaluating the risk of periodontitis, with consistent linear associations and model stability superior to HEI-2020, aMED, and DII. The lack of a focused HEI-2020 assessment framework, contradictory association between DII and periodontitis, and cultural limitations of aMED all underscored the necessity of population-specific validation for dietary pattern scoring indices. While dietary modifications showed contribution second to sex and ethnicity, our findings cautions against overreliance on inflammation-centric or regionally-biased indices, and emphasizes the heterogeneity of people with different characteristics. These results supported the prioritization of DASH in the risk assessment of periodontitis, while advocating for longitudinal designs to clarify causal relationships between diet and periodontitis, and optimizing relevant preventive strategies.

## Data Availability

Publicly available datasets were analyzed in this study. This data can be found: https://wwwn.cdc.gov/nchs/nhanes/Defa-ult.aspx.

## References

[1] Peres MA Macpherson LMD Weyant RJ Daly B Venturelli R Mathur MR. Oral diseases: a global public health challenge. Lancet. (2019) 394:249–60. doi: 10.1016/s0140-6736(19)31146-8 31327369

[2] Brauchle F Noack M Reich E. Impact of periodontal disease and periodontal therapy on oral health-related quality of life. Int Dent J. (2013) 63:306–11. doi: 10.1111/idj.12042, PMID: 24716244 PMC9375002

[3] Wu CZ Yuan YH Liu HH Li SS Zhang BW Chen W. Epidemiologic relationship between periodontitis and type 2 diabetes mellitus. BMC Oral Health. (2020) 20:204. doi: 10.1186/s12903-020-01180-w, PMID: 32652980 PMC7353775

[4] Hajishengallis G. Interconnection of periodontal disease and comorbidities: evidence, mechanisms, and implications. Periodontol. (2022) 89:9–18. doi: 10.1111/prd.12430, PMID: 35244969 PMC9018559

[5] Khajavi A Radvar M Moeintaghavi A. Socioeconomic determinants of periodontitis. Periodontol. (2022) 90:13–44. doi: 10.1111/prd.12448, PMID: 35950737

[6] Cecoro G Annunziata M Iuorio MT Nastri L Guida L. Periodontitis, low-grade inflammation and systemic health: a scoping review. Medicina. (2020) 56. doi: 10.3390/medicina56060272, PMID: 32486269 PMC7353850

[7] Wu Q Zhang W Lu Y Li H Yang Y Geng F. Association between periodontitis and inflammatory comorbidities: the common role of innate immune cells, underlying mechanisms and therapeutic targets. Int Immunopharmacol. (2024) 128:111558. doi: 10.1016/j.intimp.2024.111558, PMID: 38266446

[8] Sabbah W Tsakos G Sheiham A Watt RG. The role of health-related behaviors in the socioeconomic disparities in oral health. Soc Sci Med. (2009) 68:298–303. doi: 10.1016/j.socscimed.2008.10.030, PMID: 19027214

[9] Oberoi SS Sharma G Oberoi A. A cross-sectional survey to assess the effect of socioeconomic status on the oral hygiene habits. J Indian Soc Periodontol. (2016) 20:531–42. doi: 10.4103/0972-124x.201629, PMID: 29242690 PMC5676336

[10] Wang P Song M Eliassen AH Wang M Fung TT Clinton SK. Optimal dietary patterns for prevention of chronic disease. Nat Med. (2023) 29:719–28. doi: 10.1038/s41591-023-02235-5, PMID: 36914892 PMC10294543

[11] Padin AC Hébert JR Woody A Wilson SJ Shivappa N Belury MA. A proinflammatory diet is associated with inflammatory gene expression among healthy, non-obese adults: can social ties protect against the risks? Brain Behav Immun. (2019) 82:36–44. doi: 10.1016/j.bbi.2019.07.031, PMID: 31356923 PMC6800628

[12] Román GC Jackson RE Gadhia R Román AN Reis J. Mediterranean diet: the role of long-chain ω-3 fatty acids in fish; polyphenols in fruits, vegetables, cereals, coffee, tea, cacao and wine; probiotics and vitamins in prevention of stroke, age-related cognitive decline, and Alzheimer disease. Rev Neurol (Paris). (2019) 175:724–41. doi: 10.1016/j.neurol.2019.08.005, PMID: 31521398

[13] Yu X Pu H Voss M. Overview of anti-inflammatory diets and their promising effects on non-communicable diseases. Br J Nutr. (2024) 132:898–918. doi: 10.1017/s0007114524001405, PMID: 39411832 PMC11576095

[14] El-Sharkawy H Aboelsaad N Eliwa M Darweesh M Alshahat M Kantarci A. Adjunctive treatment of chronic periodontitis with daily dietary supplementation with omega-3 fatty acids and low-dose aspirin. J Periodontol. (2010) 81:1635–43. doi: 10.1902/jop.2010.090628, PMID: 20572767

[15] Martinon P Fraticelli L Giboreau A Dussart C Bourgeois D Carrouel F. Nutrition as a key modifiable factor for periodontitis and Main chronic diseases. J Clin Med. (2021) 10:197. doi: 10.3390/jcm10020197, PMID: 33430519 PMC7827391

[16] Shams-White MM Pannucci TE Lerman JL Herrick KA Zimmer M Meyers Mathieu K. Healthy eating Index-2020: review and update process to reflect the dietary guidelines for Americans,2020-2025. J Acad Nutr Diet. (2023) 123:1280–8. doi: 10.1016/j.jand.2023.05.015, PMID: 37201748 PMC10524328

[17] Fung TT Rexrode KM Mantzoros CS Manson JAE Willett WC Hu FB. Mediterranean diet and incidence of and mortality from coronary heart disease and stroke in women. Circulation. (2009) 119:1093–100. doi: 10.1161/circulationaha.108.816736, PMID: 19221219 PMC2724471

[18] Fung TT Chiuve SE McCullough ML Rexrode KM Logroscino G Hu FB. Adherence to a DASH-style diet and risk of coronary heart disease and stroke in women. Arch Intern Med. (2008) 168:713–20. doi: 10.1001/archinte.168.7.713, PMID: 18413553

[19] Shivappa N Steck SE Hurley TG Hussey JR Hébert JR. Designing and developing a literature-derived, population-based dietary inflammatory index. Public Health Nutr. (2014) 17:1689–96. doi: 10.1017/s1368980013002115, PMID: 23941862 PMC3925198

[20] Eke PI Thornton-Evans GO Wei L Borgnakke WS Dye BA Genco RJ. Periodontitis in US adults: National Health and nutrition examination survey 2009-2014. J Am Dent Assoc. (2018) 149:576–588.e6. doi: 10.1016/j.adaj.2018.04.023, PMID: 29957185 PMC8094373

[21] Wright DM McKenna G Nugent A Winning L Linden GJ Woodside JV. Association between diet and periodontitis: a cross-sectional study of 10,000 NHANES participants. Am J Clin Nutr. (2020) 112:1485–91. doi: 10.1093/ajcn/nqaa266, PMID: 33096553

[22] Hou K Song W He J Ma Z. The association between non-high-density lipoprotein cholesterol to high-density lipoprotein cholesterol ratio (NHHR) and prevalence of periodontitis among US adults: a cross-sectional NHANES study. Sci Rep. (2024) 14:5558. doi: 10.1038/s41598-024-56276-y, PMID: 38448487 PMC10918089

[23] Zhan JJ Hodge RA Dunlop AL Lee MM Bui L Liang D. Dietaryindex: a user-friendly and versatile R package for standardizing dietary pattern analysis in epidemiological and clinical studies. Am J Clin Nutr. (2024) 120:1165–74. doi: 10.1016/j.ajcnut.2024.08.021, PMID: 39182618 PMC11600030

[24] Eke PI Page RC Wei L Thornton-Evans G Genco RJ. Update of the case definitions for population-based surveillance of periodontitis. J Periodontol. (2012) 83:1449–54. doi: 10.1902/jop.2012.110664, PMID: 22420873 PMC6005373

[25] Tonetti MS Greenwell H Kornman KS. Staging and grading of periodontitis: framework and proposal of a new classification and case definition. J Periodontol. (2018) 89:S159–s172. doi: 10.1002/jper.18-0006, PMID: 29926952

[26] Botelho J Machado V Proença L Mendes JJ. The 2018 periodontitis case definition improves accuracy performance of full-mouth partial diagnostic protocols. Sci Rep. (2020) 10:7093. doi: 10.1038/s41598-020-63700-6, PMID: 32341429 PMC7184582

[27] Seyedhoseinpour A Barzin M Mahdavi M Valizadeh M Azizi F Ghareh S. BMI category-specific waist circumference thresholds based on cardiovascular disease outcomes and all-cause mortality: Tehran lipid and glucose study (TLGS). BMC Public Health. (2023) 23:1297. doi: 10.1186/s12889-023-16190-w, PMID: 37407928 PMC10324109

[28] Verdecchia P Reboldi G Angeli F. The 2020 International Society of Hypertension global hypertension practice guidelines – key messages and clinical considerations. Eur J Intern Med. (2020) 82:1–6. doi: 10.1016/j.ejim.2020.09.001, PMID: 32972800

[29] Takao T Suka M Nishikawa M Yanagisawa H Ishii T. Patterns of trajectories of glycated hemoglobin, fasting plasma glucose, and body mass index until the first clinic visit: the real-world history of type 2 diabetes using repeated health checkup data of Japanese workers. Fam Pract. (2025) 42:54. doi: 10.1093/fampra/cmae054, PMID: 39446604 PMC11809247

[30] Grunkemeier GL Jin R. Receiver operating characteristic curve analysis of clinical risk models. Ann Thorac Surg. (2001) 72:323–6. doi: 10.1016/s0003-4975(01)02870-3, PMID: 11515859

[31] Desquilbet L Mariotti F. Dose-response analyses using restricted cubic spline functions in public health research. Stat Med. (2010) 29:1037–57. doi: 10.1002/sim.3841, PMID: 20087875

[32] Altun E Walther C Borof K Petersen E Lieske B Kasapoudis D. Association between dietary pattern and periodontitis-a cross-sectional study. Nutrients. (2021) 13:4167. doi: 10.3390/nu13114167, PMID: 34836422 PMC8621734

[33] Perez V Chang ET. Sodium-to-potassium ratio and blood pressure, hypertension, and related factors. Adv Nutr. (2014) 5:712–41. doi: 10.3945/an.114.006783, PMID: 25398734 PMC4224208

[34] Yue Y Hovey KM LaMonte MJ Wactawski-Wende J Andrews CA Millen AE. Association between dietary patterns and periodontal disease: the OsteoPerio cohort study. J Clin Periodontol. (2024) 51:863–73. doi: 10.1111/jcpe.13979, PMID: 38538208 PMC11182713

[35] Iwasaki M Ennibi OK Bouziane A Erraji S Lakhdar L Rhissassi M. Association between periodontitis and the Mediterranean diet in young Moroccan individuals. J Periodontal Res. (2021) 56:408–14. doi: 10.1111/jre.12833, PMID: 33381869

[36] Zaragoza-Martí A Cabañero-Martínez MJ Hurtado-Sánchez JA Laguna-Pérez A Ferrer-Cascales R. Evaluation of Mediterranean diet adherence scores: a systematic review. BMJ Open. (2018) 8:e019033. doi: 10.1136/bmjopen-2017-019033, PMID: 29478018 PMC5855302

[37] Machado V Botelho J Viana J Pereira P Lopes LB Proença L. Association between dietary inflammatory index and periodontitis: a cross-sectional and mediation analysis. Nutrients. (2021) 13:1194. doi: 10.3390/nu13041194, PMID: 33916342 PMC8066166

[38] Reis RA Stolf CS de Carvalho Sampaio HA da Costa Silva BY Özlü T Kenger EB. Impact of dietary inflammatory index on gingival health. J Periodontol. (2024) 95:550–62. doi: 10.1002/jper.23-0292, PMID: 38152036

[39] Choi SW Sreeja SR Le TD Shivappa N Hebert JR Kim MK. Association between inflammatory potential of diet and periodontitis disease risks: results from a Korean population-based cohort study. J Clin Periodontol. (2023) 50:952–63. doi: 10.1111/jcpe.13817, PMID: 37085969

[40] Syrjäläinen S Männistö S Könönen E Pussinen P Gürsoy M Suominen AL. Dietary inflammatory index in relation to salivary cytokine concentrations and periodontitis: a cross-sectional analysis. J Clin Periodontol. (2024) 51:406–16. doi: 10.1111/jcpe.13917, PMID: 38158626

[41] Feng J Jin K Dong X Qiu S Han X Yu Y. Association of Diet-Related Systemic Inflammation with periodontitis and tooth loss: the interaction effect of diabetes. Nutrients. (2022) 14:4118. doi: 10.3390/nu14194118, PMID: 36235769 PMC9572370

[42] Li A Chen Y Schuller AA van der Sluis LWM Tjakkes GHE. Dietary inflammatory potential is associated with poor periodontal health: a population-based study. J Clin Periodontol. (2021) 48:907–18. doi: 10.1111/jcpe.13472, PMID: 33899265 PMC8251843

[43] Berg Y Gabay E Božić D Shibli JA Ginesin O Asbi T. The impact of nutritional components on periodontal health: a literature review. Nutrients. (2024) 16:3901. doi: 10.3390/nu16223901, PMID: 39599688 PMC11597335

[44] O'Grady J Shanahan F. Macronutrients, microbiome and precision nutrition. Curr Opin Gastroenterol. (2021) 37:145–51. doi: 10.1097/mog.0000000000000705, PMID: 33315791

[45] Worthington HV MacDonald L Poklepovic Pericic T Sambunjak D Johnson TM Imai P. Home use of interdental cleaning devices, in addition to tooth brushing, for preventing and controlling periodontal diseases and dental caries. Cochrane Database Syst Rev. (2019) 4:Cd012018. doi: 10.1002/14651858.CD012018.pub2, PMID: 30968949 PMC6953268

