# Supplementary material for: Comparison of the association between different dietary pattern scoring indices and periodontitis and their population heterogeneity
Source: Front Nutr. 2025 Jun 19;12:1590694. doi: 10.3389/fnut.2025.1590694 (PMC12224441; doi:10.3389/fnut.2025.1590694)
Supplement: Supplementary file 1 [file Supplementary_file_1.pdf]

**Supplementary materials**

**Sup. Table 1 The matrix of correlation coefficients for the four commonly used dietary pattern scoring indices of the study participants**

| Variables | HEI-2020 index | aMED index | DASH index | DII index |
|---|---|---|---|---|
| HEI-2020 index | 1.000 | --- | --- | --- |
| aMED index | 0.761 | 1.000 | --- | --- |
| DASH index | 0.788 | 0.789 | 1.000 | --- |
| DII index | -0.500 | -0.532 | -0.490 | 1.000 |

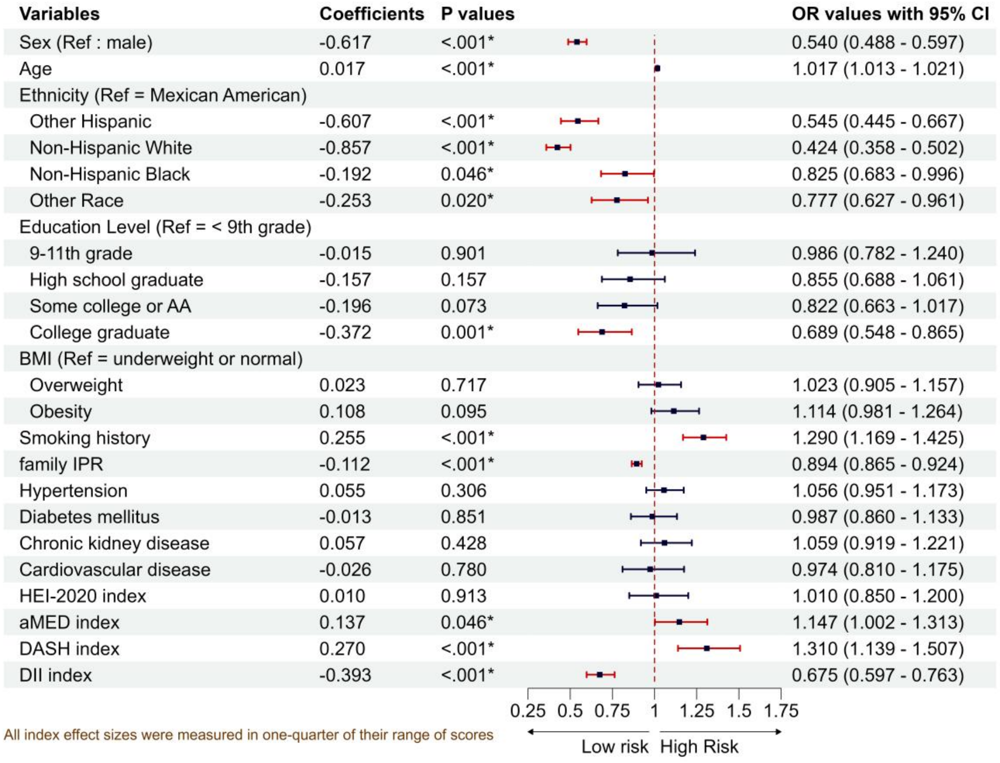


**Sup. Figure 1. The effects of all dietary pattern scoring indices and covariates on periodontitis**

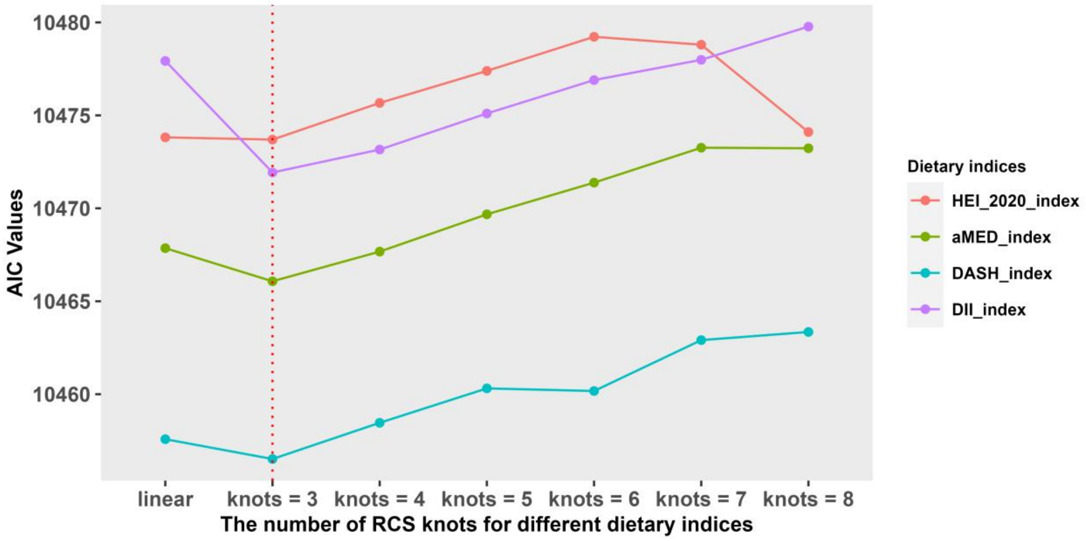


**Sup. Figure 2. The AIC values of nonlinear association models of dietary pattern indices at different knots**

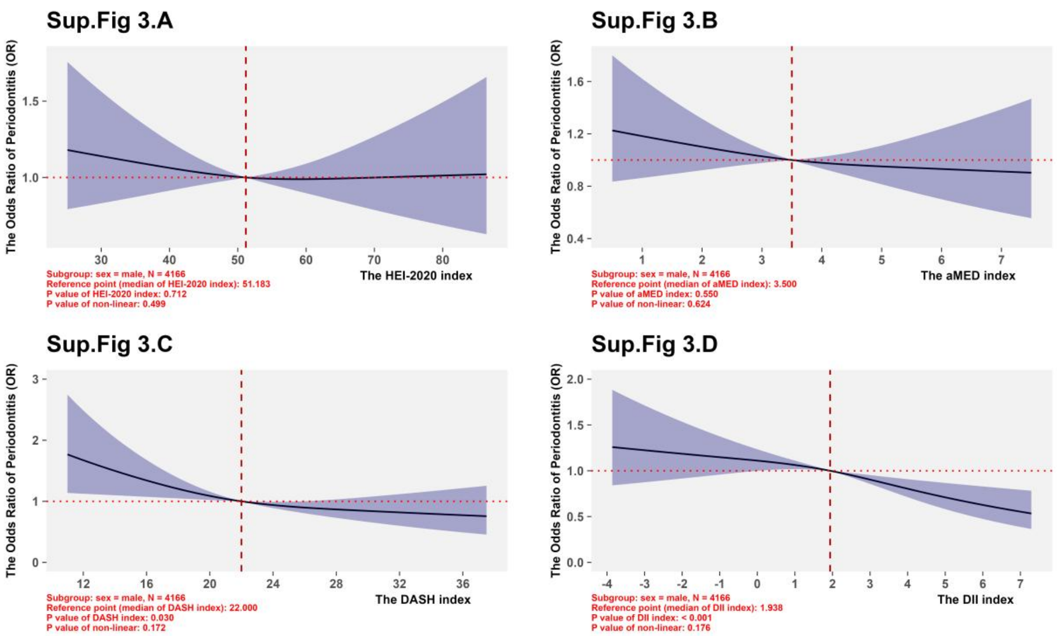


**Sup. Figure 3. Nonlinear associations between four dietary pattern scoring indices and periodontitis in the male subset**

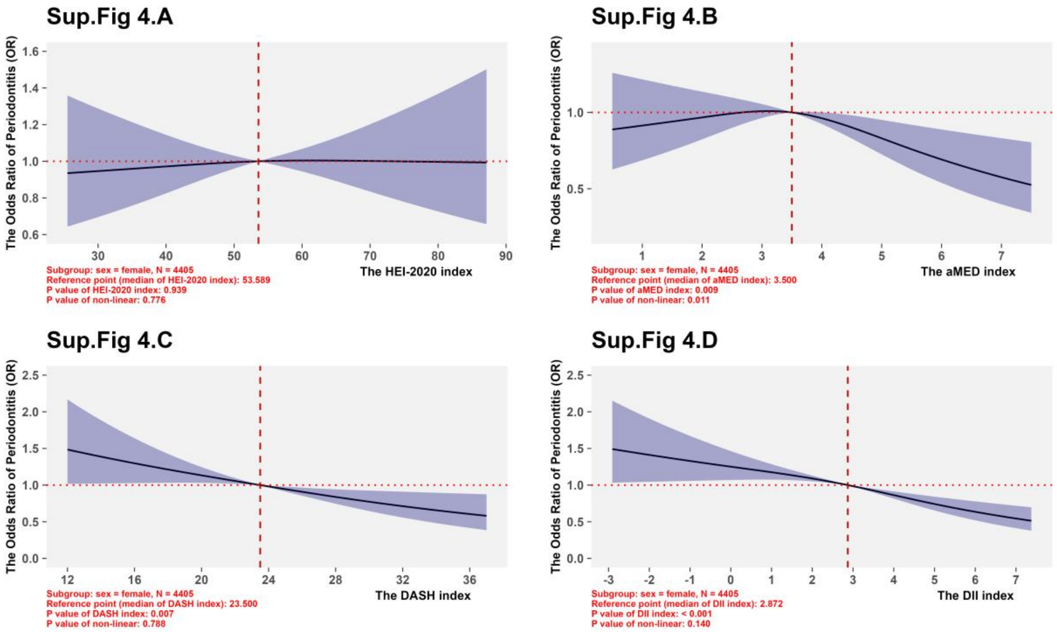


**Sup. Figure 4. Nonlinear associations between four dietary pattern scoring indices and periodontitis in the female subset**

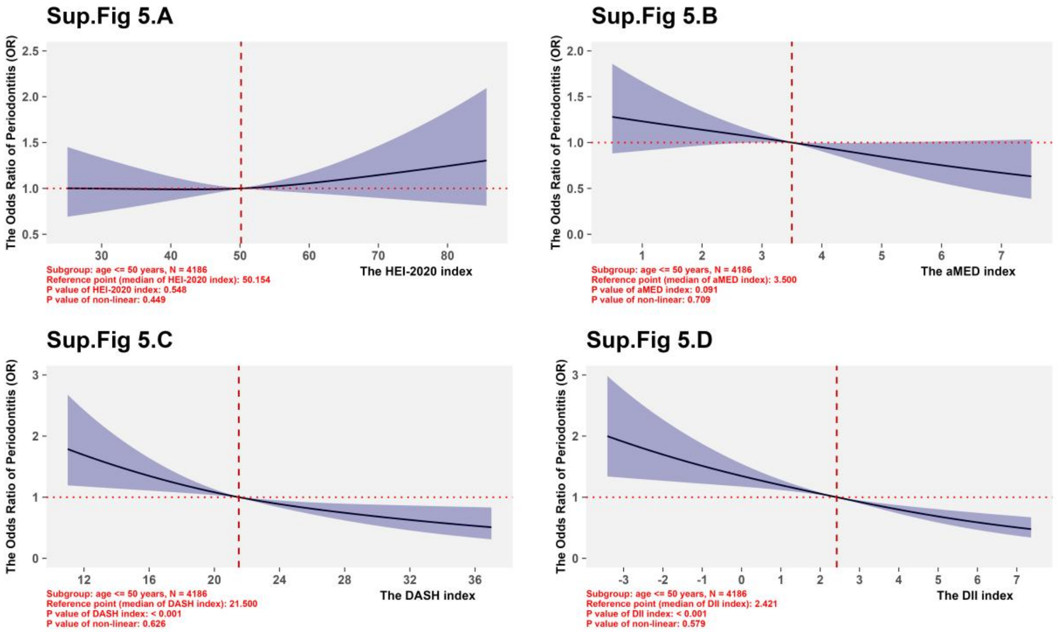


**Sup. Figure 5. Nonlinear associations between four dietary pattern scoring indices and periodontitis in the subset of participants' age under or equal to 50 years old**

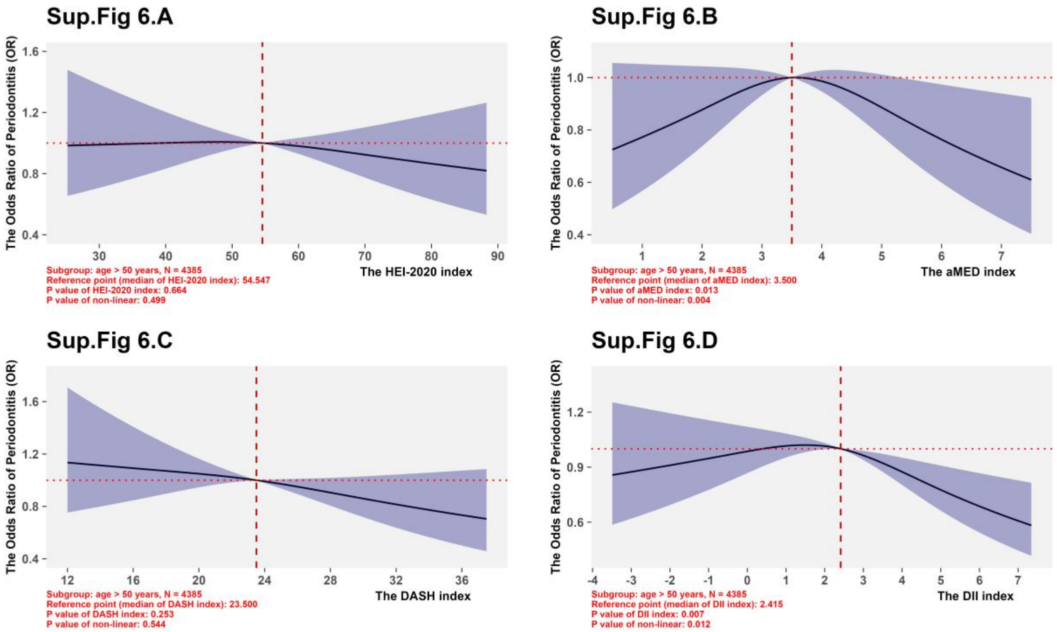


**Sup. Figure 6. Nonlinear associations between four dietary pattern scoring indices and periodontitis in the subset of participants' age over 50 years old**

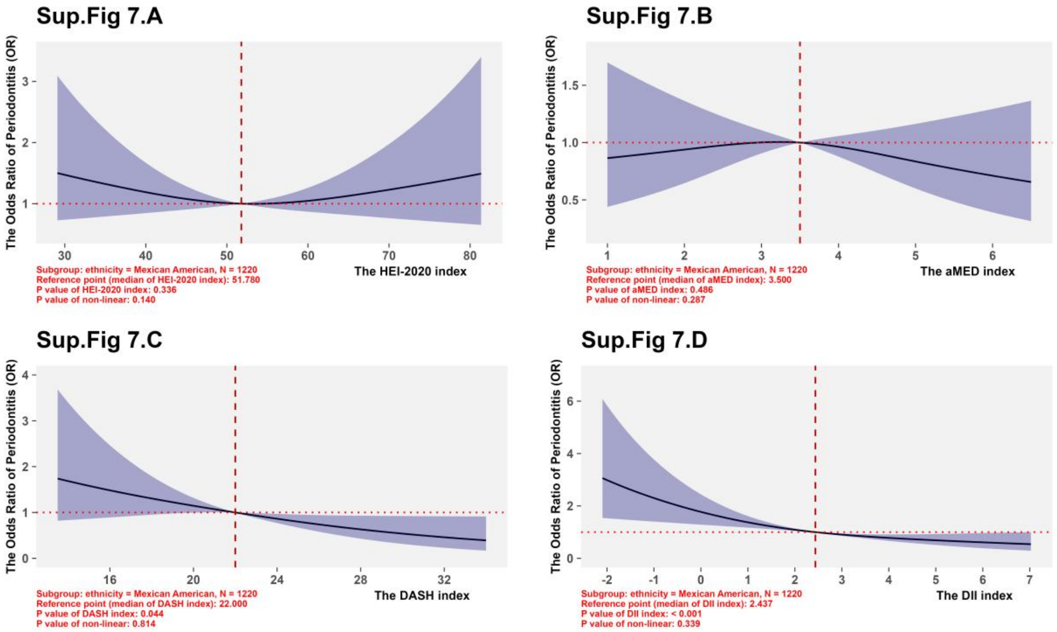


**Sup. Figure 7. Nonlinear associations between four dietary pattern scoring indices and periodontitis in the subset of Mexican American participants**

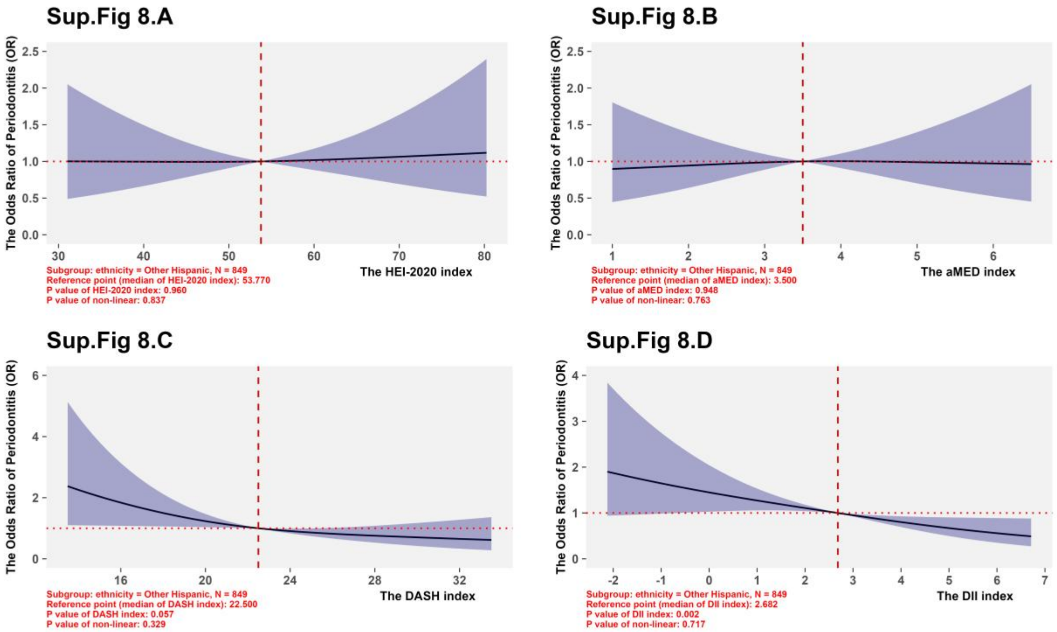


**Sup. Figure 8. Nonlinear associations between four dietary pattern scoring indices and periodontitis in the subset of Other Hispanic participants**

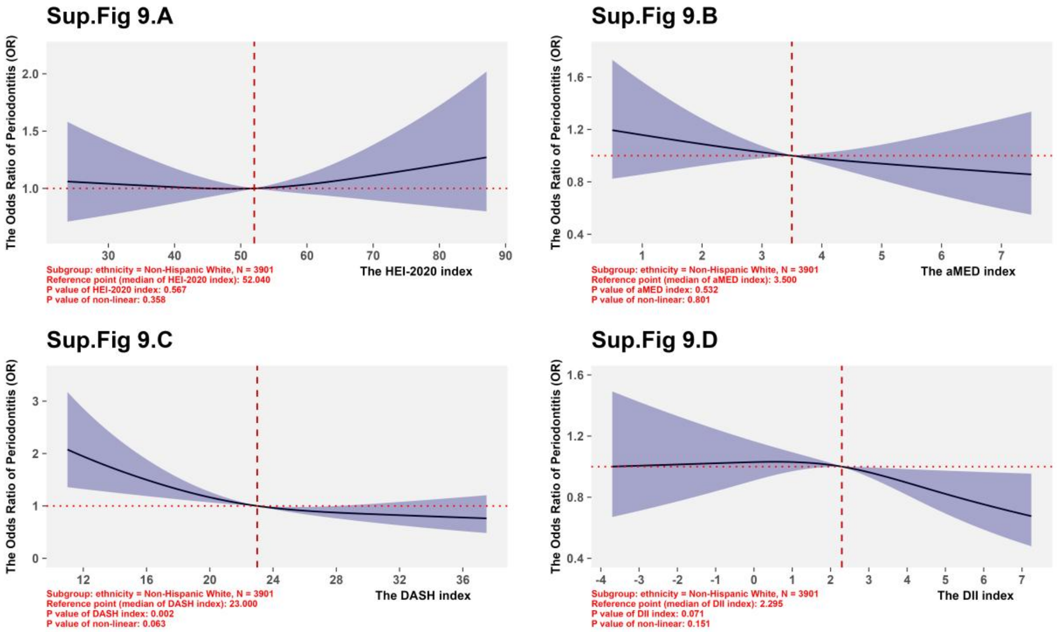


**Sup. Figure 9. Nonlinear associations between four dietary pattern scoring indices and periodontitis in the subset of Non Hispanic White participants**

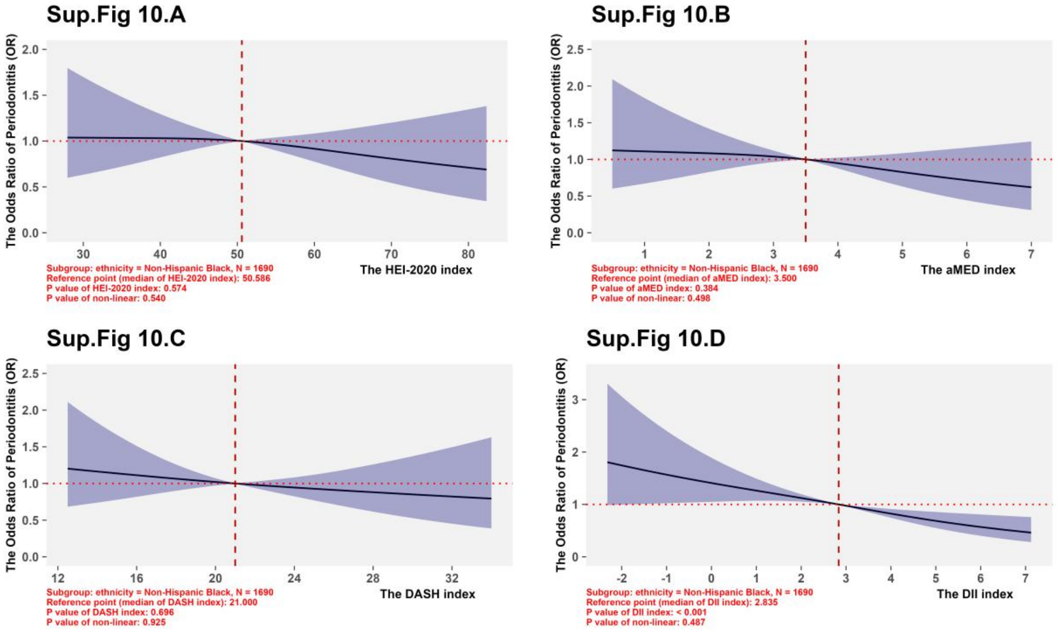


**Sup. Figure 10. Nonlinear associations between four dietary pattern scoring indices and periodontitis in the subset of Non Hispanic Black participants**

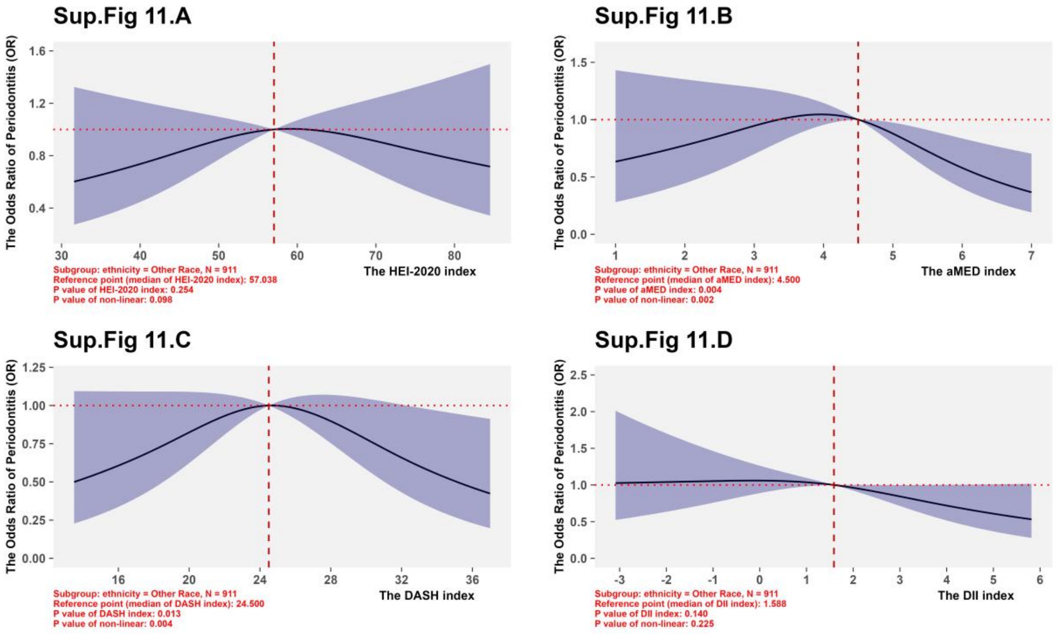


**Sup. Figure 11. Nonlinear associations between four dietary pattern scoring indices and periodontitis in the subset of Other Race participants**

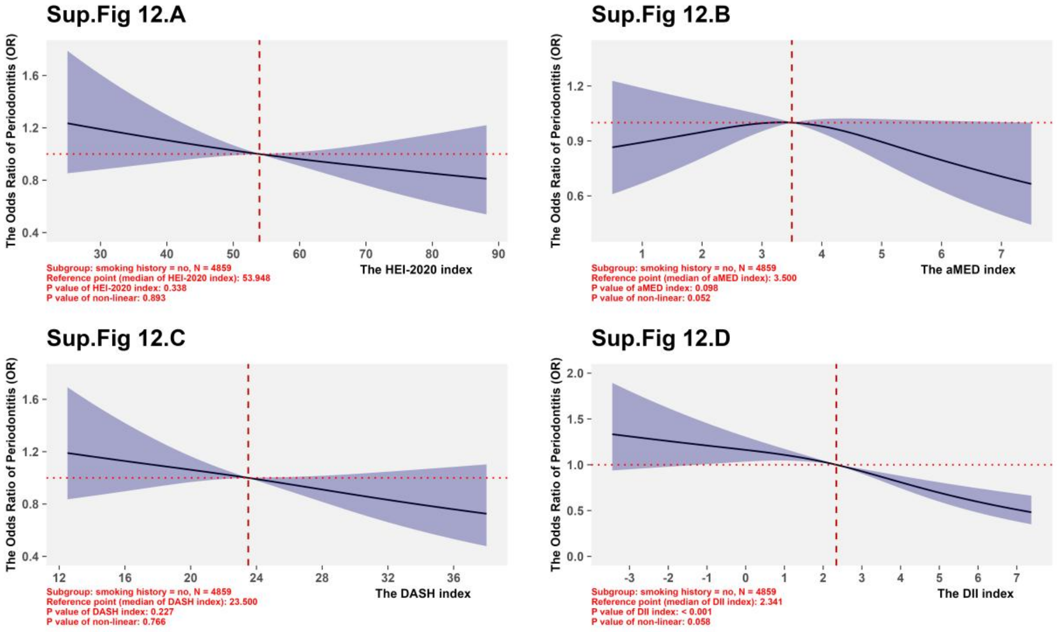


**Sup. Figure 12. Nonlinear associations between four dietary pattern scoring indices and periodontitis in the subset of no smoking participants**

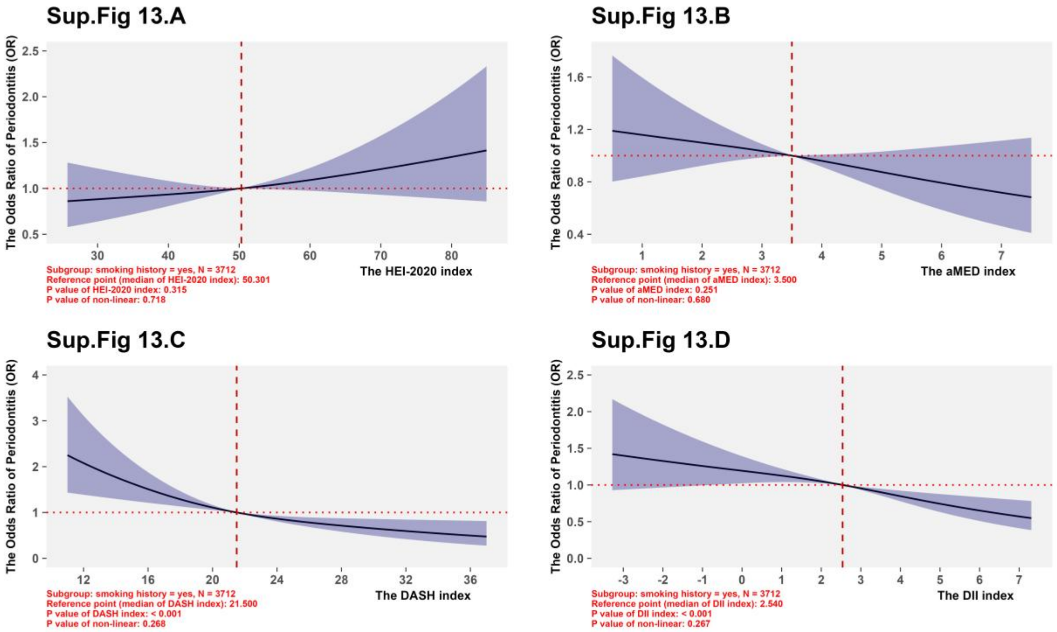


**Sup. Figure 13. Nonlinear associations between four dietary pattern scoring indices and periodontitis in the subset of smoking participants**

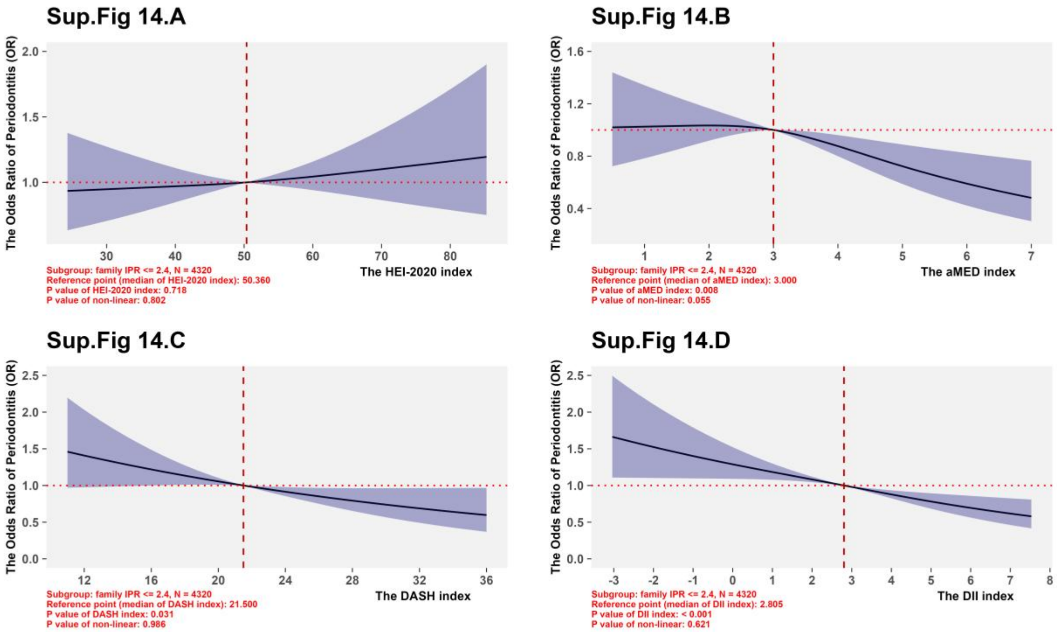


**Sup. Figure 14. Nonlinear associations between four dietary pattern scoring indices and periodontitis in the subset of participants' family IPR under or equal to 2.4**

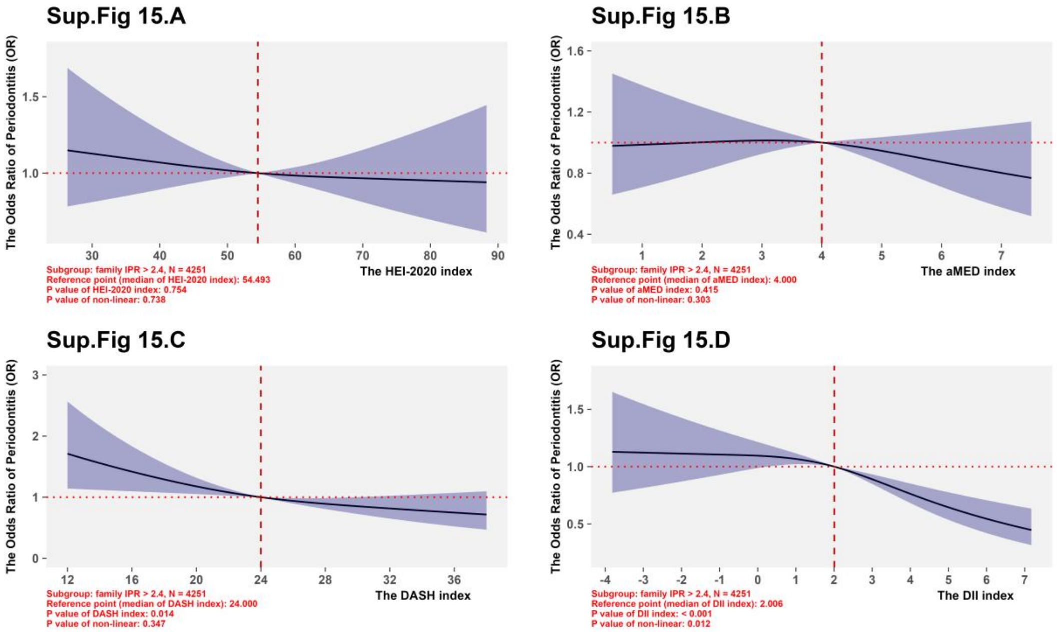


**Sup. Figure 15. Nonlinear associations between four dietary pattern scoring indices and periodontitis in the subset of participants' family IPR over 2.4**
